# Letter to the editor regarding: “Challenging unverified assumptions in causal claims: Do gas stoves increase risk of pediatric asthma?”

**DOI:** 10.1016/j.gloepi.2024.100172

**Published:** 2024-10-19

**Authors:** Kari C. Nadeau, Yannai Kashtan, Metta Nicholson, Colin J. Finnegan, Zutao Ouyang, Anchal Garg, Eric D. Lebel, Sebastian T. Rowland, Drew R. Michanowicz, Robert B. Jackson

**Affiliations:** a T.H. Chan School of Public Health, Harvard University, 677 Huntington Ave., Boston, MA 02115, USA; b PSE Healthy Energy, 1140 Broadway, Suite 750, Oakland, CA 94612, USA; c Earth System Science Department, Stanford University 473 Via Ortega, Stanford, CA 94305, USA; d College of Forestry, Wildlife and Environment, 602 Duncan Drive, Auburn, AL 36849, USA; e Woods Institute of the Environment and Precourt Institute for Energy, Stanford, CA 94305, USA

We respond here to Cox's commentary on our 2024 study titled “Nitrogen dioxide exposure, health outcomes, and associated demographic disparities due to gas and propane combustion by U.S. stoves” [1]. None of his critiques undermine the conclusions of our paper, that NO_2_ exposure from gas stoves causes thousands of pediatric asthma cases in the U.S. In particular, established principles of epidemiology call for evaluating multiple lines of evidence when assessing whether exposure to a pollutant contributes to or causes a particular health outcome [2,3]. Cox ignores these principles in his critique [4].

The deleterious effect of outdoor NO_2_ exposure on respiratory health has been well-established for decades and confirmed by numerous large meta-analyses [[5], [6], [7], [8], [9], [10], [11]]. Government agencies including the U.S. Environmental Protection Agency [[12], [13], [14]], Health Canada [13], and the European Environment Agency [15] as well as the World Health Organization [14] all agree that NO_2_ exposure is harmful generally to the respiratory system. Many studies have further shown that NO2 forms indoors in direct proportion to the amount of gas burned by a given stove [1,16,17]. We discuss Cox's specific critiques in this context of well-established harm from breathing NO_2_.

Cox's three core claims are as follows:

1) Cox claims that neither our paper nor its references established causality between NO_2_ exposure and pediatric asthma.
2) Cox claims that the relative risks we use in our model are unjustified.
3) Cox claims that the dose-response relationship we use is unjustified and untested.

We address each claim in turn.

**Causality** Cox claims that “the Kashtan et al. paper nor its reference analyze, let alone establish, causality. They do not show that reducing NO_2_ exposures from gas stoves would have any effect on pediatric asthma risk… These claims and the explicitly causal claim in the abstract are not warranted, but arise from the too-common practice of unjustifiably conflating association (in this case, based on unverified modeling assumptions, as discussed below) and causation” [4].

Merely pointing out an association between two phenomena would be insufficient to claim causality, but this is not what we did. Our causal claims are built on a decades-long body of research. Epidemiologists assess causality by reviewing the weight of evidence garnered from complementary fields, rather than by focusing only on a single type of experiment [3,18]. This framework for causality was set out by Sir Bradford Hill in the mid 1960s [19], was refined and updated in the decades since [20,21], and has since been adopted by the U.S. EPA when setting the air quality standards cited in our original paper [22]. Howick et al. grouped Hill's criteria for causality into three categories: 1) direct evidence, such as randomized control trials and properly controlled observational trials, 2) mechanistic evidence, such as laboratory studies on animals or tissues showing a direct effect of the pollutant in line with the health outcome in question, and 3) parallel evidence, such as the number of similar studies conducted and agreement among studies [20].

By these standards, the evidence for causality between NO_2_ exposure and asthma is very strong. We have 1) direct evidence: controlled laboratory studies have consistently observed adverse respiratory effects in human asthmatics when inhaling NO_2_ for short time periods compared with control groups [23] and observational studies consistently find associations between NO_2_ and pediatric asthma on a population level, both outdoors and indoors [[5], [6], [7], [8], [9], [10], [11]], [24,25].

We have 2) mechanistic evidence: laboratory studies on lung cells and mice have directly observed cell death and asthma-like symptoms following NO_2_ exposure [26,27]. The authors of one study note that “relatively short-term (24 h) exposure to NO_2_ causes epithelial damage, reduced mucin expression, and increased tone of respiratory smooth muscle [which is associated with asthma]” [26].

We have 3) parallel evidence: direct and mechanistic evidence for the effect of NO_2_ on lung function comes from dozens of studies conducted over decades [14]. Finally, we have evidence of the effect of policies that reduce NO_2_ exposure from gas stoves: our original study cited Gould et al.'s policy analysis of stove electrification in Ecuador, which found that respiratory hospitalization rates fell among participants in the electrification program, but not among those who continued to use gas [28]. The association was statistically significant and came from data covering 9.5 million hospitalizations over six years as 10 % of all Ecuadorian households electrified their cooking [28].

Based on a careful review of the evidence, the U.S. EPA's 2016 Integrated Science Assessment concludes that there is “a causal relationship between respiratory effects and short-term NO_2_ exposure, primarily based on evidence for asthma exacerbation,” that there “is likely to be a causal relationship between long-term NO_2_ exposure and respiratory effects, based primarily on evidence integrated across disciplines for a relationship with asthma development in children,” and that evidence is suggestive of a causal relationship between NO_2_ exposure and total mortality [12]. Health Canada comes to the same conclusions [13]. The European Environment Agency reports that in 2021 “52,000 deaths were attributable to NO_2_ concentrations above the WHO's guideline level of 10μg m^-3^,” implying causality between NO_2_ exposure and respiratory mortality [15]. Our original study [1] cited the exposure guidelines adopted by both U.S. and Canadian health agencies. Our study thus built on these well established, complementary lines of research to address the causality between NO_2_ exposure and asthma.

**Relative Risks** Cox claims that the relative risk values we used in our model are unjustified for two reasons:

1) The “associations studied may not even exist” because the *p*-values for the associations between NO_2_ and pediatric asthma and between gas stoves and pediatric asthma were slightly greater than 0.05. Stated another way, the 95 % confidence intervals of the relative risks overlapped with 1.

2) The meta-analyses we used may have residual confounding.

Claim 1 Statistical Significance and *p*-values Cox is arguing here that there is uncertainty in statistical associations, that there is a norm of aiming for less than 5 % uncertainty (a *p*-value of <0.05), that the associations we cite have more than this 5 % uncertainty, and that because of this the “associations studied may not even exist.”

As noted by the American Statistical Association (ASA), the *p* < 0.05 cutoff that Cox uses is arbitrary, is largely a function of study size, and ignores potentially more important factors such as how large the association is (effect size), how well-crafted the study or studies are, and what degree of uncertainty is appropriate for the association in question [29].

To examine the evidence for an association provided by the meta-analyses we cite, we'll use Puzzolo et al. [25] as an example. Puzzolo et al. calculated an association between pediatric asthma and gas stoves (expressed as an “odds ratio”) with a *p*-value of 0.07 based on an analysis of 20 studies [25]. This *p*-value is close to the common *p* < 0.05 cutoff used to assess statistical significance. Moreover, the effect size Puzzolo et al. find is large. They find that children who live in a house with a gas stove are 9 % more likely to develop asthma than those who live with an electric stove. Puzzolo et al. consider each underlying study's risk of confounding variables and incorporate this risk into their resulting estimates. Their study follows decades of research showing associations between gas stoves and pediatric asthma [12] as well as direct evidence of the toxicity of NO_2_ to lung cells [27].

Cox criticizes our analysis for employing associations whose *p*-values are (slightly) greater than 0.05. However, he does not mention that even by his own evidentiary standards (*p* < 0.05) Puzzolo et al. *did* find statistically significant associations between gas stoves and asthma in high-income countries (*p* = 0.04) such as the United States after analyzing 20 separate studies. Puzzolo et al. also found statistically significant associations between gas stoves and pneumonia (*p* = 0.025) and gas stoves and chronic obstructive pulmonary disease or COPD (*p* = 0.0011) [25]. The associations with pneumonia and COPD further strengthen the conclusion that NO_2_ exposure harms lung health.

Claim 2 Confounding Variables The meta-analysis performed by Lin et al. does find a statistically significant (*p* < 0.05) association between gas stoves and pediatric asthma [24], so Cox refers to Lin et al.'s concern of residual (i.e., unaddressed) confounding bias [4]. First, we note that many of the studies Lin et al. selected do adjust for likely confounders, such as smoking status. [24]. Lin et al. note that they “used effect estimates from the included studies which were almost always adjusted for known determinants of childhood asthma” [24]. Moreover, neither Cox nor Lin et al. identify any uncontrolled confounding pathways that could produce a positive bias. Thus it is infeasible to assess the direction of any such confounding (i.e., confounding could go in the opposite direction of the observed conditional association) nor its magnitude (e.g., could the confounding be strong enough to explain away the observed conditional association). Furthermore, to conclude that we know nothing about the respiratory harms of NO_2_ requires both ignoring other sources of scientific evidence and placing an impossible standard on observational studies.

**Our Dose-Response Model** Cox claims that “Kashtan et al. simply assume a mathematical model that implies that burden of adverse health effects is approximately proportional to exposure… They do not justify this strong assumption” [4]. Cox further claims that “Kashtan et al. provide a section on ‘Uncertainty’ that focuses solely on sampling variability, without addressing or quantifying uncertainty about the assumed model, Eq. [1] [reproduced below]” [4]. In plain English, Cox is claiming that we assumed without evidence that exposure to NO_2_ is roughly proportional to pediatric asthma risk––the more exposure, the more risk––and that we ignored the implicit uncertainty built into this assumption.

(1) $$\mathrm{Burden}={\mathrm{Inc}}_{g}\Sigma_{n}P_{n}{\mathrm{xW}}_{n}x\left(1-e^{-\beta \Delta c_{n}}\right)$$

Contrary to Cox's claim, we in fact cite three [7,30,31] epidemiological studies to justify our assumption that NO_2_ exposure is proportional to pediatric asthma risk. We model a log-linear relationship, which is the adopted standard for both the EPA and the epidemiological community [32]. This log-linear relationship is based on the scientific understanding of biochemistry, namely that as one is exposed to increasing concentrations of a toxin, the toxin's per-unit effect decreases [33]. As summarized by Anenberg et al. [6], and as we note in our original paper [1] there is some disagreement in the NO_2_ literature about whether the relationship is fully linear or log-linear and whether there is a no-effect threshold (a level of exposure at which there is no adverse effect, as suggested by Cox). For this reason, as we stated in our original paper, “we performed an analysis of the sensitivity of our calculated adverse health outcome burdens to the choice of concentration-response model… The observed differences between models were modest and statistically indistinguishable” [1]. In other words, we supported our log-linear model with the established literature and demonstrated that our findings hold whether the relationship between NO_2_ and pediatric asthma is linear or log-linear and whether there is a 2-ppb no-effect threshold or not. Readers may refer to fig. S13 in our original manuscript for our comparison of a linear model, a log-linear model, and a log-linear model with a 2 ppb no-effect threshold [1].

**Contextualizing Cox's Critiques** Combining evidence from distinct types of studies is what gives the scientific community and regulatory agencies confidence that NO_2_ causes pediatric asthma. In contrast with this comprehensive approach, Cox's rebuttal uses the inescapable limitations of certain types of studies to cast doubt on the field's findings entirely. This method of casting doubt on a scientific consensus is not new and has been used to challenge the science on smoking [34], silica dust [35], particulate matter [36], lead [37], chlorofluorocarbons [38], climate change [39], beryllium and more [40]. Our conclusions – that NO2 exposure from gas stoves causes asthma – stand.

## Funding

This research did not receive any specific grant from funding agencies in the public, commercial, or not-for-profit sectors*.*

## CRediT authorship contribution statement

**Kari C. Nadeau:** Writing – review & editing, Supervision, Conceptualization. **Yannai Kashtan:** Writing – review & editing, Writing – original draft, Conceptualization. **Metta Nicholson:** Writing – review & editing. **Colin J. Finnegan:** Writing – review & editing, Conceptualization. **Zutao Ouyang:** Writing – review & editing. **Anchal Garg:** Writing – review & editing. **Eric D. Lebel:** Writing – review & editing. **Sebastian T. Rowland:** Writing – review & editing, Conceptualization. **Drew R. Michanowicz:** Writing – review & editing, Conceptualization. **Robert B. Jackson:** Writing – review & editing, Supervision, Conceptualization.

## Declaration of competing interest

The authors declare no competing interests.

## References

[1] Kashtan Y., Nicholson M., Finnegan C.J., Ouyang Z., Garg A., Lebel E.D. (2024). Nitrogen dioxide exposure, health outcomes, and associated demographic disparities due to gas and propane combustion by U.S. stoves. Sci Adv.

[2] Dominici F., Goldman G.T. (2019). Don’t abandon evidence and process on air pollution policy. Science.

[3] Richmond-Bryant J. (2020). In defense of the weight-of-evidence approach to literature review in the integrated science assessment. Epidemiology.

[4] Cox L.A. (2024). Challenging unverified assumptions in causal claims: do gas stoves increase risk of pediatric asthma?. Glob Epidemiol.

[5] Khreis H., Kelly C., Tate J., Parslow R., Lucas K., Nieuwenhuijsen M. (2017). Exposure to traffic-related air pollution and risk of development of childhood asthma: a systematic review and meta-analysis. Environ Int.

[6] Anenberg S.C., Henze D.K., Tinney V., Kinney P.L., Raich W., Fann N. (2018). Estimates of the global burden of ambient PM2.5, ozone, and NO2 on asthma incidence and emergency room visits. Environ Health Perspect.

[7] Achakulwisut P., Brauer M., Hystad P., Anenberg S.C. (2019). Global, national, and urban burdens of paediatric asthma incidence attributable to ambient NO2 pollution: estimates from global datasets. Lancet Planet. Health.

[8] Zhang Z., Wang J., Lu W. (2018). Exposure to nitrogen dioxide and chronic obstructive pulmonary disease (COPD) in adults: a systematic review and meta-analysis. Environ Sci Pollut Res.

[9] Huangfu P., Atkinson R. (2020). Long-term exposure to NO2 and O3 and all-cause and respiratory mortality: a systematic review and meta-analysis. Environ Int.

[10] Hoffmann C., Maglakelidze M., von Schneidemesser E., Witt C., Hoffmann P., Butler T. (2022). Asthma and COPD exacerbation in relation to outdoor air pollution in the metropolitan area of Berlin, Germany. Respir Res.

[11] Chen Z., Liu N., Tang H., Gao X., Zhang Y., Kan H. (2022). Health effects of exposure to sulfur dioxide, nitrogen dioxide, ozone, and carbon monoxide between 1980 and 2019: a systematic review and meta-analysis. Indoor Air.

[12] US Environmental Protection Agency (2018). Review of the primary National Ambient air Quality Standards for oxides of nitrogen. https://cfpub.epa.

[13] Health Canada (2016).

[14] World Health Organization (2021). https://apps.who.int/iris/handle/10665/345329?locale-attribute=ar&s=.

[15] European Environment Agency “Harm to human health from air pollution in Europe: burden of disease 2023” (Briefing). https://www.eea.europa.eu/publications/harm-to-human-health-from-air-pollution/harm-to-human-health-from.

[16] Lebel E.D., Finnegan C.J., Ouyang Z., Jackson R.B. (2022). Methane and NOx emissions from natural gas stoves, cooktops, and ovens in residential homes. Environ Sci Technol.

[17] Singer B.C., Pass R.Z., Delp W.W., Lorenzetti D.M., Maddalena R.L. (2017). Pollutant concentrations and emission rates from natural gas cooking burners without and with range hood exhaust in nine California homes. Build Environ.

[18] Morabia Alfredo (2024).

[19] Bradford S.A., Cbe H., Frcp D. (1965). Frs, the environment and disease: association or causation?. Proc R Soc Med.

[20] Howick J., Glasziou P., Aronson J.K. (2009). The evolution of evidence hierarchies: what can Bradford Hill’s ‘guidelines for causation’ contribute?. J R Soc Med.

[21] Fedak K.M., Bernal A., Capshaw Z.A., Gross S. (2015). Applying the Bradford Hill criteria in the 21st century: how data integration has changed causal inference in molecular epidemiology. Emerg Themes Epidemiol.

[22] “National Ambient Air Quality Table” (US Environmental Protection Agency (2023). https://www.epa.gov/criteria-air-pollutants/naaqs-table.

[23] Brown J.S. (2015). Nitrogen dioxide exposure and airway responsiveness in individuals with asthma. Inhal Toxicol.

[24] Lin W., Brunekreef B., Gehring U. (2013). Meta-analysis of the effects of indoor nitrogen dioxide and gas cooking on asthma and wheeze in children. Int J Epidemiol.

[25] Puzzolo E., Fleeman N., Lorenzetti F., Rubinstein F., Li Y., Xing R. (2024). Estimated health effects from domestic use of gaseous fuels for cooking and heating in high-income, middle-income, and low-income countries: a systematic review and meta-analyses. Lancet Respir Med.

[26] Hussain I., Jain V.V., O’Shaughnessy P., Businga T.R., Kline J. (2004). Effect of nitrogen dioxide exposure on allergic asthma in a murine model. Chest.

[27] Persinger R.L., Blay W.M., Heintz N.H., Hemenway D.R., Janssen-Heininger Y.M.W. (2001). Nitrogen dioxide induces death in lung epithelial cells in a density-dependent manner. Am J Respir Cell Mol Biol.

[28] Gould C.F., Bejarano M.L., De La Cuesta B., Jack D.W., Schlesinger S.B., Valarezo A. (2023). Climate and health benefits of a transition from gas to electric cooking. Proc Natl Acad Sci.

[29] Wasserstein R.L., Lazar N.A. (2016). The ASA statement on statistical significance and P-values. Am Stat.

[30] Anenberg S.C., Mohegh A., Goldberg D.L., Kerr G.H., Brauer M., Burkart K. (2022). Long-term trends in urban NO2 concentrations and associated paediatric asthma incidence: estimates from global datasets. Lancet Planet Health.

[31] Mohegh A., Goldberg D., Achakulwisut P., Anenberg S.C. (2020). Sensitivity of estimated NO2-attributable pediatric asthma incidence to grid resolution and urbanicity. Environ Res Lett.

[32] Sacks J.D., Lloyd J.M., Zhu Y., Anderton J., Jang C.J., Hubbell B. (2018). The Environmental Benefits Mapping and Analysis Program – Community Edition (BenMAP–CE): A tool to estimate the health and economic benefits of reducing air pollution. Environ. Model. Softw..

[33] Yahyaeian A.E., Shahidi M., Mousavi T., Daniali M., Wexler P. (2024). Encyclopedia of toxicology.

[34] Brandt A.M. (2012). Inventing conflicts of interest: a history of tobacco industry tactics. Am J Public Health.

[35] Michales D. (2020). Triumph of Doubt.

[36] Michaels D. (2020). Triumph of Doubt.

[37] Markowitz G., Rosner D. (2013).

[38] Oreskes N., Conway E. (2011). Merchants of doubt.

[39] Oreskes N., Conway E. (2011).

[40] Michaels D., Proctor R., Schiebinger L. (2008). “Manufactured uncertainty” in Agnotology: The making and unmaking of ignorance.

